# Smartphone Addiction and Interpersonal Competence of Nursing Students

**Published:** 2018-03

**Authors:** Sunhee LEE, Hye-Jin KIM, Han-Gyo CHOI, Yang Sook YOO

**Affiliations:** 1. Dept. of Nursing, College of Nursing, Catholic University of Korea, Seoul, Korea; 2. Dept. of Nursing, College of Medicine, University of Ulsan, Ulsan, Korea

**Keywords:** Interpersonal competence, Smartphone addiction, Nursing student

## Abstract

**Background:**

Interpersonal competence is an important capacity for nurses. Recently, the advent of smartphones has instigated considerable changes in daily life. Because smartphone has multiple functions, people tend to use them for numerous activities, often leading to addictive behavior.

**Methods:**

This cross-sectional study performed a detailed analysis of smartphone addiction subscales and social support related to interpersonal competence of nursing students. Overall, 324 college students were recruited at Catholic University in Seoul, Korea from Feb 2013 to Mar 2013. Participants completed a self-reported questionnaire, which included scales that measured smartphone addiction, social support, interpersonal competence, and general characteristics. Path analysis was used to evaluate structural relations between subscales of smartphone addictions, social support, and interpersonal competence.

**Results:**

The effect of cyberspace-oriented relationships and social support on interpersonal competence were 1.360 (*P*=.004) and 0.555 (*P*<.001), respectively.

**Conclusion:**

Cyberspace-oriented relationship, which is a smartphone addiction subscale, and social support were positively correlated with interpersonal competence of nursing students, while other smartphone addiction subscales were not related to nursing student interpersonal competence. Therefore, effective smartphone teaching methods be developed to enhance nursing student motivation

## Introduction

As the healthcare environment changes, responsibilities, required competencies, and skill expectations for nurses are increasing. Therefore, nurse educators deliver curricula that meet the new, diverse current health service demands (1). Some nursing researchers have examined the required competencies for nursing students (1, 2). Internationally, competency-based curricula are universally accepted as the most appropriate strategy for teaching and assessing student performance (1). The International Council of Nurses (ICN) has defined competence as “a level of performance of the task with desirable outcomes under the varied circumstances of the real world” (3). Competence is essential in undergraduate nursing curricula. However, there is wide disparity incompetence definitions in the nursing context (4). Interpersonal competence (IC) of nurses is important (1, 2). Nevertheless, it is not easy to find research on nurse IC. Therefore, it is necessary to evaluate nursing student IC in order to use that information to develop nursing education curricula.

IC is defined as social interaction within close personal relationships and includes initiating relationships, self-disclosure, providing emotional support, asserting displeasure for others’ actions, and managing interpersonal conflicts (5). Research in the IC area suggests that individuals with an interpersonal competence are more likely to build relationship networks that provide support in the face of stressful life events (6). IC is related to social support (7) as well as adolescent friendship (8). With regard to IC nursing research, studies investigating communication skills have been conducted. Cancer patients and those who care for them often suffer psychological stress, reduced by effective communication and support from their attending physician, nurse, or another healthcare professional (6).

In addition, development of internet-based smart devices has made on innovative impact on human society (9). Addictive people tend to feel depressed and isolated without their smartphone, and smartphone addiction-like behavior is a serious problem related to loss of control (10). Self-control behaviors intend to enhance the long-term interests of the individual. People exert self-control when they follow rules or inhibit immediate desires to delay gratifications or intention implementation (11). Control over impulsive behavior can be used proactively to counteract the influence of short-term outcomes (12). Loss of control can affect school adjustment including academic and social adjustment (13). Smartphones are already a crucial part of many users’ lives. It might be difficult for smartphone users to focus on their work because they cannot get their smartphone off their minds (9). Excessive smartphone use can cause behavioral difficulties for studying, relationship with others, and IC for users including nursing students, therefore, nursing students can have trouble in concentrating on their studies due to smartphone use, especially during school hours. In addition, college students are required to self-regulate behaviors compared to middle- and high school students. College student smartphone addiction can result in loss of control and affect social adjustment, including IC. However, nursing researchers have not investigated the effect of smartphone addiction in nursing students.

Thus, the present cross-sectional study aimed to analyze smartphone addiction and social support related to nursing student IC.

## Materials and Methods

### Study design

We conducted a descriptive, exploratory study using path analysis to examine the relationships between smartphone addiction subscales, social support, and nursing student IC.

### Setting and sample

Participants were nursing students enrolled in a baccalaureate program at a university in Seoul, Korea. All student levels (freshmen, sophomore, junior, and senior) were invited to participate. Overall, 324 students participated, and participants were recruited from Feb 2013 to Mar 2013. Participants completed a self-report questionnaire, which included scales that measured smartphone addiction, social support, IC, and general characteristics.

### Ethical approval

This study was approved by the institutional review board (IRB) of the university (No. MC13QISI0008). Participants were informed of the study purpose and their right to refuse to participate without penalty. Students who wanted to participate in the study were asked to read and sign the informed consent. Finally, the researcher distributed and collected the questionnaires.

### Measurements

#### Smartphone addiction inventory (SAI)

The smartphone addiction inventory (SAI), developed for Korean university students (14), was used to measure smartphone addiction. We selected this scale because it considers cultural differences. Because smartphone use is closely related to daily life, the SAI might be helpful in properly investigating smartphone addiction in Korean university students. The SAI consists of 23 items using a five-point Likert-type scale (1 as ‘totally disagree’ to 5 as ‘totally agree’). The SAI subscales are preoccupation (six items), daily-life disturbance (five items), withdrawal (four items), overuse (six items), and cyber-oriented relationships (two items). Total scores range from 23 to 115, with higher scores indicating a higher level of smartphone addiction. The reliability coefficient (Cronbach’s α) for the SAI in the original study on 201 Korean university students was 0.86 (14), and the reliability coefficient in this study was 0.87.

#### Multidimensional scale of perceived social support (MSPSS)

The multidimensional scale of perceived social support (MSPSS) was used to measure social support. It was developed (15) and translated into Korean (16). It consists of 12 items using a seven-point Likert-type scale (1 as ‘very strongly disagree’ to 7 as ‘very strongly agree’). The MSPSS has three subscales containing four items that identify support from family, friends, and significant others. Total score ranges from 12 to 84, with higher scores indicating a higher level of perceived social support. The reliability coefficient for MSPSS in a study of 275 university students was 0.85 (15), and the reliability coefficient in the present study was 0.95.

#### Korean version of the IC questionnaire (K-ICQ)

The K-ICQ (17) was used to measure IC. The K-ICQ was revised from the original ICQ, developed (5), using translation and reverse translation. Although the ICQ comprises 40 items and 5 subscales, the K-ICQ consists of 31 items using a five-point Likert-type scale (1 as ‘totally disagree’ to 5 as ‘totally agree’) and five subscales determined via exploratory factor analysis. The sub-scales are initiation (eight items), assertion (seven items), caring for others (seven items), conflict management (six items), and appropriate disclosure (three items). Three factors (initiation, assertion, and conflict management) were similar to the original ICQ, but the other two (caring for others and appropriate disclosure) were added to represent the unique interpersonal culture in Korea. Possible K-ICQ scores range from 31 to 155, with higher scores indicating a higher IC level. The reliability coefficient for ICQ in a study of 422 college students was 0.87 (5). The reliability coefficient for the K-ICQ in a study of 182 university students was 0.90 (17), whereas the reliability coefficient for the K-ICQ in the present study was 0.92.

#### Data collection

After IRB approval, data were collected from Feb 2013 to Mar 2013. We visited lecture rooms at the end of class to distribute questionnaires. Before distribution, we explained the purpose of the study, that there was no requirement to participate, and that participation had no influence on school life or grades. Questionnaire was distributed and collected directly by two trained research assistants. The questionnaires were completed in 15–25 min.

#### Data analyses

Descriptive statistics were generated using SPSS (ver. 20 Chicago, IL, USA). Independent sample t-tests and an analysis of variance (ANOVA) were performed to identify significant differences in participant IC level by general characteristics. Path analysis was generalized to evaluate the structural relationships between smartphone addiction, social support, and IC variables derived from the respective subscale scores. The structural model for path analysis was verified by structural equation modeling (SEM) using AMOS 20.0, and model fit was assessed using comparative fit index (CFI), normed fit index (NFI), Tucker-Lewis Index (TLI), and root mean square error of approximation (RMSEA). As mentioned above, smartphone addiction consisted of five sub-concepts of preoccupation, daily-life disturbance, withdrawal, overuse, and cyber-oriented relationships. We tried to investigate how each sub-concept affected IC considering that cyber-oriented relationships can affect IC differently compared to the other sub-concepts. In addition, social support can strongly affect nursing student IC; therefore, we tried to analyze using path analysis without any mediators.

## Results

The majority of participants were female (91.4%) and younger than 25 yr (98.1%). There were 83 freshmen (25.6%), 73 sophomores (22.5%), 87 juniors (26.9%), and 81 seniors (25.0%). Of the total, 189 (58.3%) participants reported they followed a religion, and 256 (79.0%) perceived their economic status as average. There were no significant differences between nursing student IC values based on general characteristics (Table 1).

**Table 1:** General characteristics of nursing students and the associated levels of interpersonal competence

***Variables***	***N (%)***	***Interpersonal competence***	
		**M (SD)**	**T or F (p)**
Gender			
Male	28 (8.6)	100.64 (15.95)	0.97 (0.33)
Female	296 (91.4)	97.46 (16.42)	
Age (yr)			
≤20	181 (55.9)	96.66 (16.63)	0.95 (0.39)
21–25	137 (42.3)	99.20 (15.23)	
≥26	6 (1.9)	96.83 (31.11)	
Grade			
Freshmen	83 (25.6)	98.53 (17.70)	1.71 (0.16)
Sophomore	73 (22.5)	94.85 (16.09)	
Junior	87 (26.9)	96.79 (15.77)	
Senior	81 (25)	100.53 (15.65)	
Religion			
Yes	189 (58.3)	99.05 (17.33)	2.93 (0.09)
No	135 (41.7)	95.90 (14.81)	
Perceived economic status			
Good	25 (7.7)	104.76 (17.21)	2.53 (0.08)
Average	256 (79)	97.22 (16.77)	
Bad	43 (13.3)	96.72 (12.45)	

N = 324

The average IC score was 97.73 (range: 51–145). Among the five subscales, “initiation” showed the highest mean score (24.78), whereas “appropriate disclosure” showed the lowest mean score (8.30). In addition, the average smartphone addiction score was 47.83 (range: 23–85). Among the five subscales, “overuse” and “cyberspace-oriented relationship” showed the highest (12.44) and the lowest (3.40) mean scores, respectively. The average social support score was 67.79 (range: 24–84). Among the three subscales, “significant others” showed the highest mean score (22.79), whereas “family” (22.38) showed the lowest (Table 2).

**Table 2:** Sub-concepts of interpersonal competence, smartphone addiction, and social support

***Category (range)***	***Mean (SD)***
Interpersonal competence (31–155)	97.73(16.38)
Initiation (8–40)	24.78(5.76)
Appropriate disclosure (3–15)	8.30(2.51)
Care for others (7–35)	23.85(4.09)
Assertion (7–35)	20.76(5.11)
Conflict management (6–30)	20.04(3.69)
Smartphone addiction (23–115)	47.83 (11.95)
Preoccupation (6–30)	11.60 (3.62)
Daily-life disturbance (5–25)	10.05 (3.14)
Withdrawal (4–20)	10.32 (3.69)
Overuse (6–30)	12.44 (3.40)
Cyberspace-oriented relationship (2–10)	3.40 (1.85)
Social support (12–84)	67.79 (10.12)
Family (4–28)	22.38 (3.99)
Friends (4–28)	22.62 (3.56)
Significant others (4–28)	22.79 (3.56)

N = 324

**Fig. 1:**
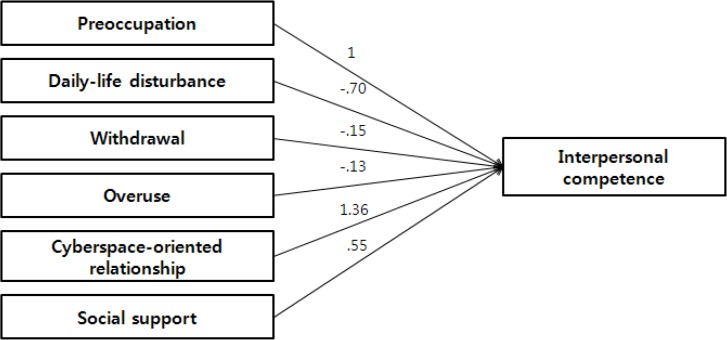
The estimated path coefficients of factors that influence IC

A path model was developed to examine variables that affect nursing student IC. The analyses indicated a good model fit for all indices (χ^2^ = 1.954, CFI = 0.998, NFI = 0.997, TLI = 0.957, RMSEA = 0.054). Fig. 1 and Table 3 present the estimated path coefficients for factors that influence IC. Among the factors, the effects of cyberspace-oriented relationships and social support on IC were 1.360 and 0.555, respectively. An effect is considered small, moderate, or large, when the absolute path coefficient value is less than 0.10, between 0.10 and 0.50, and greater than 0.50, respectively. Thus, cyberspace-oriented relationships and social support appeared to have large effect on nursing student IC.

**Table 3:** Analysis of factors influencing interpersonal competence

***Latent variable***	***Path***	***Estimate***	***S.E.***	***C.R.***	***P***
Smartphone addiction	IC ← Preoccupation	1.000			
	IC ← Daily-life disturbance	−.700	.382	−1.831	.067
	IC ← Withdrawal	−.154	.294	−.523	.601
	IC ← Overuse	−.131	.343	−.382	.703
	IC ← Cyberspace-oriented relationship	1.360	.467	2.909	.004
Social support	IC ← Social support	.555	.088	6.284	<.001

IC: Interpersonal competence. // N = 324

However, other subscale factors of smartphone addiction (preoccupation, daily-life disturbance, withdrawal, and overuse) did not appear to affect nursing student IC.

## Discussion

The mean score of smartphone addiction and IC in this study were 47.83 and 97.73, respectively. In comparison to smartphone addiction (14) and IC (17) for university students with majors other than nursing, these smartphone addiction and IC scores are low. For example, the mean smartphone addiction score for university students in a study (14) was 52.6. The IC score for university students in a study (17) was 100.95. Smartphone addiction level in nursing students was lower than that for other university students. Nursing students in this study tended to exert self-control in order to achieve their long-term outcome, such as academic adjustment. Nursing students in this study also showed low IC level compared to another university student in a study (14). IC is a required competency for nursing students (18), therefore, we need to develop a program to improve IC for nursing students.

Our findings also indicate that cyberspace-oriented relationships and social support have a large positive effect on nursing student IC, whereas preoccupation, daily-life disturbance, withdrawal, and overuse were not associated with nursing student IC. In contrast to this study, addictive smartphone usage was related to a decrease in real life social community participation and relationship problems (19). However, smartphones can strengthen family bonds, expand psychological neighborhoods, and facilitate proximity to the people they call (20). In addition, smartphones now include social network services (SNS) such as Facebook, thus potentially enabling users to enhance interpersonal networks (21, 22). This is consistent with our finding that cyberspace-oriented relationships and social support are positively correlated in nursing students.

In addition, all means of the smartphone addiction subscales in this study were lower than the median; therefore, nursing students can be regarded as having the ability to control their smartphone use. Consequently, an important issue to consider about smartphone use in nursing education is not to prohibit smartphone use in class, but rather to use them effectively for nursing education. Smartphone applications can be a valuable tool for nursing education, and computer-based references have straightforward search functions to allow students to find topics of interest (23). Use of smartphones increases health providers’ communication efficacy (24), and encouraging smartphone use also encourages self-directed learning by allowing learners to research questions as they arise (25).

This study has two limitations. First, although the sample size was statistically adequate, participants were recruited only from one university in Korea. Thus, these results might not generalize to all nursing students. Second, the data were self-reported. Therefore, the actual interpersonal competency among nursing students could be overestimated.

## Conclusion

The cyberspace-oriented relationship, which is a smartphone addiction subscale, and social support were associated with nursing student IC, while other smartphone addiction subscales did not affect nursing student IC. Nursing students can use their smartphones to communicate with their colleagues about academic issues and to refer to websites when searching for advanced specialized knowledge. Thus, there are likely benefits to be derived from developing effective teaching methods incorporating smartphones to increase nursing student motivation. We also recommend future longitudinal or experimental studies to explore whether smartphone social networking services influence IC.

## Ethical considerations

Ethical issues (Including plagiarism, informed consent, misconduct, data fabrication and/or falsification, double publication and/or submission, redundancy, etc.) have been completely observed by the authors.
